# Changes in patient-centered attitude and confidence in communicating with patients: a longitudinal study of resident physicians

**DOI:** 10.1186/s12909-018-1129-y

**Published:** 2018-01-25

**Authors:** Hirono Ishikawa, Daisuke Son, Masato Eto, Kiyoshi Kitamura, Takahiro Kiuchi

**Affiliations:** 1 Department of Health Communication, School of Public Health, The University of Tokyo, 7-3-1, Hongo, Bunkyo-ku, Tokyo, 113-8655 Japan; 2 International Research Center for Medical Education, Graduate School of Medicine, The University of Tokyo, 7-3-1, Hongo, Bunkyo-ku, Tokyo, 113-0033 Japan; 3 General Education Center, University of Tokyo Hospital, 7-3-1, Hongo, Bunkyo-ku, Tokyo, 113-0033 Japan; 4 School of Medicine, International University of Health and Welfare, 4-3 Kozunomori, Narita-shi, Chiba, 286-8686 Japan

**Keywords:** Communication skills, Gender, Patient-centeredness, Postgraduate education

## Abstract

**Background:**

Patient-centered care has been one of the most frequently discussed principles in medical practice. However, there is a serious concern that the patient-centered attitudes of physicians diminish over the course of their medical education. This longitudinal study examined changes in resident physicians’ patient-centered attitudes and their confidence in communicating with patients, and explored the relationship between the two traits.

**Methods:**

The study participants were resident physicians at a university hospital in Tokyo. Participants’ patient-centered attitudes (as measured by the Patient–Practitioner Orientation Scale [PPOS]), and their confidence in communicating with patients (as per the Physician Confidence in the Medical Interview scale: [PCMI]) were assessed through self-reported questionnaires completed at the beginning of residency (*n* = 204) and again at the end of the first year (*n* = 95).

**Results:**

PPOS scores declined significantly during the year, both in terms of attitude toward sharing information and decision-making with patients, and attitude of caring for patients’ expectations and emotions. The shift in caring attitude differed significantly by gender. The increase in PCMI score was greater for those with a smaller decrease in PPOS score.

**Conclusions:**

As seen in previous studies of medical students, resident physicians’ patient-centered attitudes declined during their first year of residency, while there may be a gender-based difference within the shift. The increase in physicians’ confidence in communicating with patients was greater for those who showed a smaller decline in patient-centered attitude. Additional studies are needed to detail the changes in physicians’ attitudes, confidence, and communication skills over the course of their medical training, and to develop systematic assessment and training programs.

## Background

Over the past few decades, patient-centered care has been one of the most frequently discussed principles in medical practice [1]. Two-way communication, as a fundamental means for patients and physicians to exchange views and seek common ground, is considered a central component of such care. Medical schools have instituted a host of educational approaches to foster patient-centered attitude and communication skills among medical students. Yet despite such educational efforts, there has been concern that physicians’ patient-centered attitudes may diminish over the course of their medical education [2].

However, the available evidence on this attitudinal change is still contradictory. Some studies have reported such a decline after clinical clerkship [3, 4], while other studies found either an increase or no significant decline [5, 6]. Similarly, inconsistent reports have been made on evidence related to physician empathy. A systematic review of studies on medical students and residents reported that self-perceived empathy declined significantly over the course of medical school and residency [7]. However, findings of other studies, especially those conducted outside the United States, have been contradictory [8, 9], and the validity and practical significance of the decline in empathy have been questioned [10].

Physician empathy is an important element of patient-centered communication. Moreover, this communication includes not only understanding and accommodating of patients’ perspectives and feelings, but also sharing power and information to reach a shared understanding of the problem and of treatment-related decision-making [11]. Some studies have reported a decline in sharing with an increase in caring, suggesting that changes may differ between these components of a patient-centered attitude [3, 5]. Few studies have longitudinally examined changes in patient-centered attitude within individuals, and most previous studies have focused on medical students.

Currently, in Japan, graduates of 6 years of medical school must undertake a mandatory 2-year residency for their postgraduate medical education [12]. Attitude and communication skills for sharing power and information may be more likely to be learned during this residency in which, in their interactions with patients, they serve as physicians rather than medical students. It has been reported that physicians with a more patient-centered attitude showed more attention to lifestyle issues, took less of a biomedical focus, and made greater efforts to establish rapport when communicating with patients [13]. However, it is not still clear whether the change in a physician’s attitude is related to their learning and achievement of skills for communicating with patients.

In this prospective longitudinal study, we described the trends in resident physicians’ patient-centered attitudes and their confidence in communicating with patients, and explored the relationship between attitude changes and confidence. More specifically, we pursued the following research questions.

1. Does patient-centered attitude among resident physicians decline over the first year of a residency program?
2. Does physicians’ confidence in communicating with patients increase over their first year of a residency program?
3. Is the change in physicians’ confidence in communicating with patients associated with the change in patient-centered attitude?

## Methods

### Participants and procedures

This is a longitudinal trend study. Study participants were resident physicians who began a junior residency program at a university hospital in Tokyo during the academic years of 2013–2015. They were invited to participate in the baseline questionnaire survey (named T1) during the orientation sessions just before starting the residency program. Residents were informed that participation was voluntary and would not influence how they were evaluated in their residency. Written informed consent was obtained from the participants. Among 221 resident physicians who attended the session, 204 residents (67 [93.1%] in 2013, 66 [91.7%] in 2014, and 71 [92.2%] in 2015) returned a completed consent form and questionnaire (response rate: 92.3%). At the end of the academic year, a follow-up questionnaire was sent to the T1 participants by in-house mail and online (named T2). Ninety-five out of 204 (27 [40.3%] in 2013, 41 [62.1%] in 2014, and 27 [38.0%] in 2015) returned the questionnaire (response rate: 46.6%). Each participant received a 500-yen Amazon gift certificate yen at T1 and at T2 respectively, when they returned the questionnaire.

Study procedures were approved by the Institutional Review Board of the Graduate School of Medicine, The University of Tokyo (#10075).

### Measures

#### Physicians’ patient-centered attitudes

The Patient–Practitioner Orientation Scale (PPOS) is a well-validated instrument for assessing an individual’s attitude toward the patient–physician relationship [11, 14]. A previous Japan-based study translated and validated the scale [15]. The scale contains 18 items reflecting two domains of patient-centered practice: Sharing and Caring. The Sharing subscale assesses the extent to which the individual believes the patient should receive information and be involved in decision-making, while the Caring subscale assesses the extent to which the patient’s expectations, feelings, and lifestyle should be taken into consideration during medical consultation. Scoring is based on a six-point Likert scale ranging from “strongly agree” to “strongly disagree,” with higher scores indicating a more patient-centered inclination.

### Physicians’ confidence in communicating with patients

The Physician Confidence in the Medical Interview (PCMI) scale was developed in a previous study of resident physicians, seeking to assess their confidence in communicating with patients during medical interviews [15]. The participants in the present study were asked about their confidence in achieving 21 specific behavioral objectives under seven communication tasks deemed important for a patient-centered medical interview: 1) initiating the session, 2) gathering information, 3) sharing information, 4) planning, 5) closing the session, 6) providing a structure, and 7) building a relationship. Additionally, a single item asked about their overall confidence in conducting a medical interview that the patient would consider satisfactory and acceptable. Each item was rated on a four-point Likert scale ranging from “not confident at all” to “very confident,” with higher scores indicating greater confidence in communicating with the patient.

Demographic characteristics (age, gender, and specialty orientation) were also obtained through the self-reported questionnaire at T1.

### Statistical analysis

First, differences in participant characteristics at T1 between T2 participants and non-participants were examined by chi-square test or independent t-test. Then, among T2 participants, changes in PPOS and PCMI scales were examined by paired t-test. Stratified analyses by gender were also conducted because in previous studies [3–6, 11, 16] gender was found consistently related to patient-centered attitude. The change was also calculated as T2 score minus T1 score, and the differences in change by gender were examined by analysis of covariance, controlling for the T1 score. The relationship of the change in PPOS with that in PCMI was examined by Pearson correlation. Analyses were conducted with Stata 14.2 software (StataCorp LP, College Station, TX, USA).

## Results

### Participant characteristics

Table 1 shows the characteristics of all T1 participants, and the differences between the subgroups of those who participated in T2 and those who did not. There were no statistically significant differences in the characteristics and the PPOS and PCMI scores at T1. The PPOS scores of all T1 participants were generally higher for female residents than male residents (not shown in Table 1). The differences between male and female residents were statistically significant for the total PPOS (4.50 vs. 4.63, *p* = 0.032) and the Caring subscale (4.71 vs. 4.87, *p* = 0.012), but not for the Sharing subscale (4.29 vs. 4.38, *p* = 0.219). There was no significant difference in PCMI scores (2.45 vs. 2.55, *p* = 0.189). The specialty orientation was not statistically significantly associated with the PPOS and PCMI.

**Table 1:** Participant characteristics and descriptive statistics of PPOS and PCMI scores

		T1 participants (*N* = 204)		T2 participants (*N* = 95)		T2 non-participants (*N* = 109)		
		N	%	N	%	N	%	*p*-value ^a)^
Gender	Male	112	54.9	56	59.0	56	51.4	0.278
	Female	92	45.1	39	41.1	53	48.6	
Specialty orientation	Internal	78	38.2	44	46.3	34	31.2	0.078
	Surgical	50	24.5	19	20.0	31	28.4	
	Other	38	18.6	19	20.0	19	17.4	
	undecided/missing	38	18.6	13	13.7	25	22.9	
		Mean	SD	Mean	SD	Mean	SD	
Age	(years)	26.24	3.26	25.82	2.73	26.61	3.63	0.087
PPOS		4.56	0.43	4.50	0.48	4.61	0.38	0.079
Share		4.33	0.54	4.26	0.61	4.39	0.46	0.093
Care		4.79	0.47	4.74	0.49	4.83	0.45	0.201
PCMI		2.50	0.53	2.43	0.56	2.55	0.50	0.102

*PPOS* patient–practitioner orientation scale, *PCMI* physician confidence in the medical interview

^a^Differences between T2 participants and non-participants were examined by chi-square test or t-test

### Changes in PPOS and PCMI

As shown in Table 2, the PPOS scores (both total and two subscales) were significantly lower at T2 than T1. When stratified by gender, the decline was statistically significant for the PPOS and Caring subscale for male residents, while no significant decline was observed for female residents. In contrast, the PCMI significantly improved from T1 to T2 for both males and females. The change in PPOS (T2 − T1) differed significantly by gender for the Caring subscale, controlling for the T1 score.

**Table 2:**
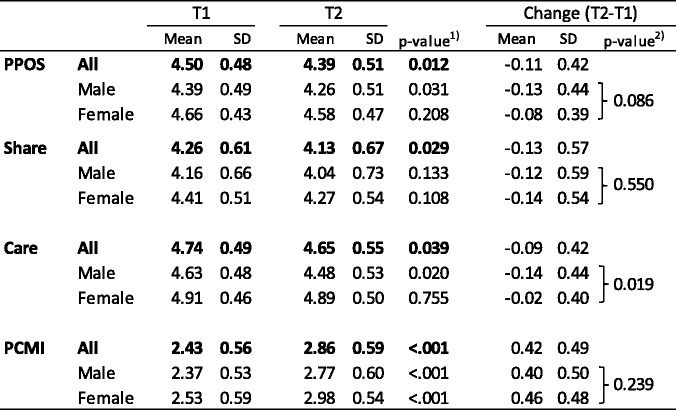
Changes of PPOS and PCMI scores between T1 and T2 by gender (*N* = 95)

*PPOS* patient–practitioner orientation scale, *PCMI* physician confidence in the medical interview

^1^) Paired t-test between T1 and T2

^2^) Analysis of covariance of the score change (T2-T1) by gender controlling for T1 score

### Relationship of changes in PPOS and PCMI

Physicians’ patient-centered attitude, as measured by the PPOS, was generally positively associated with confidence in communicating with patients as measured by the PCMI (Table 3). The association was statistically significant at the time of T1.

**Table 3:** Associations between PPOS and PCMI scores at T1, T2, and the change (T2 − T1) (*N* = 95)

	r	*p*-value
T1	0.207	0.045
T2	0.191	0.064
T2-T1	0.216	0.035

*PPOS* patient–practitioner orientation scale, *PCMI* physician confidence in the medical interview

The changes in PPOS score and in PCMI score were positively associated with each other, suggesting that the residents who had an increase in patient-centered attitude over the year achieved more greatly increased confidence in communicating with patients.

## Discussion

This study longitudinally examined the changes in resident physicians’ attitudes and confidence when communicating with patients, and explored the relationship between the changes in the two traits.

Significant declines in patient-centered attitude over the first year of their residency program were observed for both components: sharing information and decision-making with the patient, and caring about patients’ expectations and emotions. Previous studies have suggested the clinical practice phase of training and distress as main reasons for the decline in patient-centeredness and empathy [3, 7]. Undergraduate medical education in Japan have typically provided less opportunity for clinical education to interact with actual patients, compared to the US medical schools [17], although it is currently being reformed to increase clinical rotation hours in order to meet the criteria by the Educational Commission for Foreign Medical Graduates. On the other hand, it has been reported that resident physicians in Japan deliver a large volume of high-value patient care, while receiving little structured education and enduring substantial sleep deprivation [18]. Previous studies indicated that high levels of stress and burnout were related to deterioration in empathy and patient-centered communication among resident physicians [19]. The shift in patient-centered attitudes could be strongly influenced by the systems of medical education and clinical care, which should be further explored in future studies.

As has been consistently reported in previous studies [3–6, 11, 16], female residents were generally more inclined to a patient-centered attitude. The difference by gender was more evident for the caring component than the sharing component. The present findings give further evidence that the attitudinal shift in caring attitude significantly differed by gender. Male residents became less patient-centered in terms of caring attitude after 1 year, while female residents showed little decline. Further study is needed to explore how female students manage to maintain their patient-centered attitude while presumably going through comparable experiences in their medical training. A previous meta-analysis of observational studies of physician communication indicated that female physicians engaged in more communication that could be considered patient-centered, such as addressing psychosocial issues, using emotional talk and positive talk, and more actively incorporating patient input [20]. According to the Social Learning Theory proposed by Bandura [21], individuals acquire new behaviors by observing and imitating others. Those that are observed are called role models, and provide examples of behavior to observe and imitate. Characteristics of a role model are often that they are the same gender as the observer, have higher status to the observer and are older than the observer. Residents may be more likely to learn ways of interacting with patients during their residency from senior physicians of the same gender who serve as role models. Male residents may have adopted less patient-centered attitudes from their own senior male physicians who were less inclined to this approach compared with their female counterparts.

Contrary to the decline in the patient-centered attitude, residents became more confident in conducting patient-centered medical interviews over the first year of their residency. In general, those with more a patient-centered attitude were more confident in communicating with patients. The increase in such confidence was greater for those with a smaller decline in patient-centered attitude. During the residency, there are few formal mandatory educational programs for developing communication skills. Residents are largely expected to learn from their everyday interactions with patients and by observing senior physicians. What they learn during their residency likely varies depending on their attitude or motivation regarding communication with patients. Those who maintained a patient-centered attitude may have been more likely to value patient-centered communication and make efforts to learn those associated skills.

### Limitations

Our study had several limitations. First, the sample was derived from a single university hospital in Japan, which may limit the generalizability of our finding. At least, however, this hospital has one of the largest groups of residents undergoing a junior residency program, and these residents come from medical schools across the country. Second, the response rate considerably dropped at T2 (46.6%). This was partly because the questionnaire was distributed and collected by in-house mail and online at T2, unlike T1 survey that was conducted during the orientation session. Although there was no statistically significant difference in baseline characteristics between T2 participants and non-participants, the changes in patient-centered attitude and confidence in communication with patients might have been different between them. Third, sociocultural context and practice style may differ between Japanese and Western cultures, and our findings may be specific to the Japanese context. At a minimum, however, the findings related to PPOS scores were essentially consistent with previous US-based studies. Forth, it is widely acknowledged that physicians’ self-assessment of their competence does not necessarily reflect their actual competence [22, 23]. At least, however, another part of our study revealed that physicians’ confidence in communicating with patients as measured via the PCMI was related to their observed communication behaviors during a simulated medical interview [24]. Fifth, since this was an observational study, causal relationships between physicians’ attitude and confidence cannot be established.

### Implications for practice and future research

The increase in physicians’ confidence in communicating with patients was greater for those who showed a smaller decline in patient-centered attitude. Previous studies have suggested that innovative educational programs may prevent erosion of patient-centered attitudes among medical students [25, 26]. Additional studies are needed to detail the changes in physicians’ attitudes, confidence, and communication behaviors over the course of their medical training, and to develop systematic training and assessments of physicians’ patient-centered attitudes and their skills for communicating with patients. Further, the sociocultural context including communication and practice styles and educational systems should be considered in investigating how physicians’ patient-centered attitudes relate to their learning and achievement of patient-centered communication skills.

## Conclusions

In the present study, as seen in previous studies of medical students, resident physicians’ patient-centered attitudes declined during their first year of residency. The shift in caring attitude significantly differed by gender, suggesting that male residents show greater decline in this trait than female residents. The increase in physicians’ confidence in communicating with patients was greater for those with a smaller decline in their patient-centered attitude. The residency program should consider including systematic training in and assessment of communication skills. The measures of physicians’ confidence and attitudes in communicating with patients might be useful to identify individuals with greater needs and to evaluate the impact of the educational program.
